# Necrotizing enterocolitis associated with *Clostridium butyricum* in a Japanese man

**DOI:** 10.1002/ams2.329

**Published:** 2018-01-23

**Authors:** Yukio Sato, Dai Kujirai, Katsura Emoto, Toshiaki Yagami, Taketo Yamada, Manabu Izumi, Masaki Ano, Kenichi Kase, Kenji Kobayashi

**Affiliations:** ^1^ Department of Emergency Medicine Saiseikai Utsunomiya Hospital Tochigi Japan; ^2^ Department of Emergency and Critical Care Medicine Keio University School of Medicine Tokyo Japan; ^3^ Department of Pathology Saiseikai Utsunomiya Hospital Tochigi Japan; ^4^ Department of Pathology Keio University School of Medicine Tokyo Japan; ^5^ Department of Radiology Saiseikai Utsunomiya Hospital Tochigi Japan; ^6^ Department of Pathology Saitama Medical University Saitama Japan; ^7^ Department of General Internal Medicine Saiseikai Utsunomiya Hospital Tochigi Japan; ^8^ Department of Critical Care Medicine Saiseikai Utsunomiya Hospital Tochigi Japan; ^9^ Department of Surgery Saiseikai Utsunomiya Hospital Tochigi Japan

**Keywords:** Adult, Asia, *Clostridium butyricum*, enterocolitis, necrotizing

## Abstract

**Case:**

Necrotizing enterocolitis (NEC) caused by *Clostridium butyricum* is common in neonates; however, a case of NEC in adults has not been previously reported. An 84‐year‐old Japanese man developed *C. butyricum*‐related NEC during hospitalization for treatment of stab wounds to the left side of the neck and lower abdomen, without organ damage, and concomitant pneumonia.

**Outcome:**

The patient developed acute onset of emesis accompanied by shock during his admission; partial resection of the small intestine was carried out due to necrosis. Pathologic findings showed mucosal necrosis and extensive vacuolation with gram‐positive rods in the necrotic small intestine. Blood culture tests revealed *C. butyricum* infection. The patient's condition improved after the surgery. He was moved to a rehabilitation hospital on day 66.

**Conclusion:**

This study suggests that hospitalized adult patients who receive antibiotic treatment are at risk for NEC.

## Introduction

Several strains of *Clostridium butyricum* have been cultured from the stool of healthy children and adults.^1^ One of those strains, MIYAIRI 588, is used widely as a probiotic in Asia, including Japan.^2^ It has been reported that it inhibited the cytotoxicity of *Clostridium difficile* in an *in vitro* study and reduced *C. difficile* toxin A in an *in vivo* study.^3^, ^4^ However, some of these strains produce endotoxins and cause necrotizing enterocolitis (NEC) in neonates.^5^ The only toxin *C. butyricum* has been reported to produce is analogous to the type E botulinum neurotoxin secreted by *Clostridium botulinum*.^6^ After the first report of NEC due to *C. butyricum* type E in an infant,^7^ many similar cases have been reported, including two cases of intestinal botulism involving adolescents,^8^ and one case of sepsis in an adult.^9^ Additionally, cases of food‐borne botulism caused by *C. butyricum* have been reported.^10^ However, a case of NEC due to *C. butyricum* in an adult has not been reported to date.

## Case

An 84‐year‐old man visited our hospital owing to a neck (left) and abdominal penetrating injury by a short sword in a suicide attempt. The patient had a medical history of cerebral infarction and paroxysmal atrial fibrillation on apixaban. He lived in his home with his family and had no recent history of hospitalization or admission to a nursing home.

On examination, his vital signs were normal except for disturbed consciousness: Glasgow coma scale score, 6; blood pressure, 158/92 mmHg; respiratory rate, 18 breaths/min; heart rate, 72 b.p.m.; and body temperature, 35.9°C. A short sword had been inserted into the left lateral neck. There was no exit wound. There was another stab wound in the middle of the lower abdomen. There were no hard signs of bleeding around either wound. There were no peritoneal signs. Otherwise, the physical examination was unremarkable. Laboratory examination showed: white cell count, 13,000/μL; neutrophils, 11,180/μL; lymphocytes, 1,300/μL; hemoglobin, 12.3 g/dL; platelet count, 33.6 × 10^4^/μL; activated partial thromboplastin time, 27.8 s; prothrombin time – international normalized ratio, 1.27; and fibrinogen level, 377 mg/dL.

**Figure 1:**
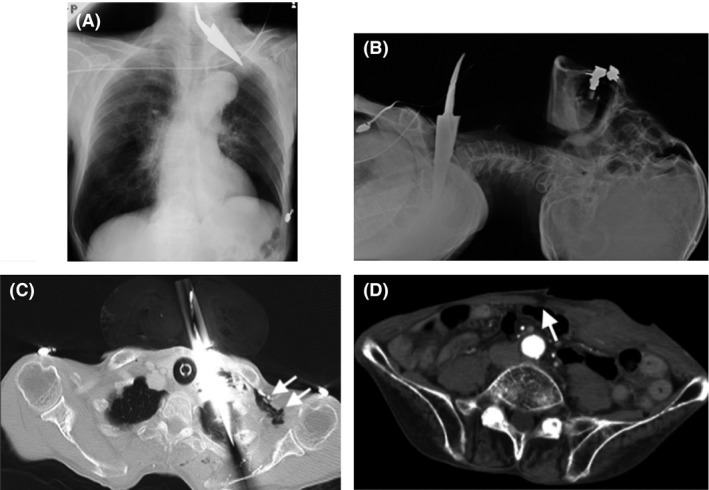
Radiographs and whole body computed tomography (CT) scans taken on admission of an 84‐year‐old man with self‐inflicted stab wounds to the neck and abdomen. A, Radiograph of the chest. B, Radiograph of the neck. C, CT image of the chest. Arrows indicate the air leakage. D, CT image of the abdomen. The arrow indicates the injury to the posterior layer of the rectus sheath.

On image examination, a chest radiograph did not indicate a hemopneumothorax, and a focused assessment with sonography for trauma did not indicate fluid in the chest or abdominal cavities (Fig. 1A). To examine the trajectory of the sword, a cervical radiograph was carried out (Fig. 1B). Neck and chest radiographs did not show any free air in the soft tissue. A whole body computed tomography (CT) scan with i.v. contrast revealed the sword penetrating through the left thoracic cavity from the left side of the neck and an injury in the lower abdomen. There was s.c. emphysema with signs of pneumothorax. The peritoneum was penetrated; however, there were no free air or free fluid in the abdominal cavity (Fig. 1C,D). It was difficult to ascertain whether the left subclavian artery was injured because of an artifact on the CT images generated by the sword; therefore, emergency exploratory thoracotomy and laparotomy were carried out.

**Figure 2:**
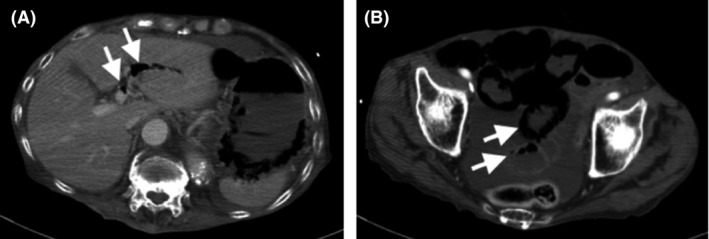
Whole body computed tomography scan of an 84‐year‐old man with self‐inflicted stab wounds to the neck and abdomen, taken when the patient presented with vomiting. A, Image of the upper abdomen. Arrows indicate portal vein gas. B, Image of the lower abdomen. Arrows indicate pneumatosis cystoides intestinalis.

**Figure 3:**
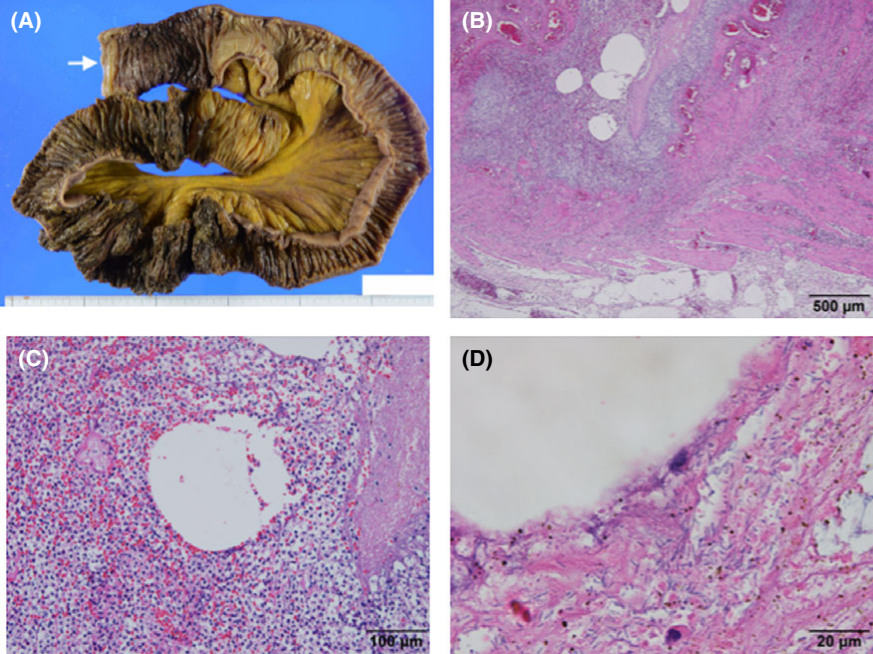
Histological observation of the resected small intestine of an 84‐year‐old man with necrotizing enterocolitis associated with *Clostridium butyricum*, using hematoxylin–eosin staining with a macro image. A, Macro image of resected tract. Arrow indicates the oral side. B, Severe inflammation, several vacuoles, congestion, and hemorrhage in the muscularis propria and subserosal layer indicate gas gangrene. Scale bar, 500 μm. C, Vacuoles are surrounded by acute severe inflammation and considered as gas accumulation. Scale bar, 100 μm. D, Observation by oil immersion lens reveals numerous bacilli adjacent to vacuoles. Scale bar, 20 μm.

During the surgery, no organ damage was found, and the sword was removed safely. The patient was treated in the intensive care unit after surgery; however, he needed continuous mechanical ventilation owing to respiratory failure. Subsequently, the patient developed ventilator‐associated pneumonia due to methicillin‐resistant *Staphylococcus aureus* on postoperative day (POD) 13 and was treated using meropenem 1.0 g/day and vancomycin 2 g/day i.v. until POD 23. The patient was extubated on POD 20. The patient was transferred from the intensive care unit and moved to the step‐down unit on POD 21. As ambulation and oral intake were difficult, due to a continuously disturbed mental state, enteral feeding was continued. On POD 36, the patient presented with sudden onset of vomiting, with hypoxemia and shock. On examination before intubation, generalized abdominal tenderness with peritoneal signs was recognized. Laboratory examination showed: white blood count, 11,240/μL; neutrophils, 10,453/μL; lymphocytes, 674/μL; hemoglobin 7.4 g/dL; platelet count, 34.5 × 10^4^/μL; activated partial thromboplastin time, 33.6 s; prothrombin time – international normalized ratio, 1.38; and C‐reactive protein, 3.21 mg/dL. Niveau formation was observed on the chest radiograph obtained after intubation. A whole‐body CT scan showed portal vein gas and pneumatosis cystoides intestinalis, suggesting ischemic enteritis (Fig. 2). Consequently, the patient was brought to the operating room for the emergent exploratory laparotomy. Despite the patient's age, arteriosclerosis suggesting ischemic enteritis was not observed. One hundred and twenty centimeters of jejunum and ileum was resected, located 70 cm away from the ligament of Treitz; the first 20 cm was necrotic, the next 30 cm looked pale, suggesting ischemia, and the last 70 cm was necrotic (Fig. 3A). Mesenterium at the region was congested with blood. The remaining intact tracts, comprising 170 cm in total, were stapled by functional end‐to‐end anastomosis. Histopathologic examination of the resected intestine showed extensive mucosal necrosis, innumerable gram‐positive bacilli, and associated vacuolation and epithelial regeneration (Fig. 3B–D). The findings suggested NEC. In addition, *C. butyricum*, a gram‐positive bacillus, was concurrently isolated from two cultured blood samples drawn just before the partial enterectomy. We also reconfirmed that the blood culture samples obtained on POD 42 were sterile. An antibiotic, meropenem 1.0 g/day, was given for 2 weeks, starting at onset of shock, and had a susceptibility to *C. butyricum*. The patient recovered and was moved to a rehabilitation hospital on POD 66 following his first surgery.

## Discussion

Necrotizing enterocolitis caused by *C. butyricum* is common in preterm neonates, and can be life‐threatening.^5^ However, to the best of our knowledge, no such cases have been reported in adults.

In the present case, we initially suspected ischemic enteritis; however, surgical and pathological findings suggested NEC. In addition to the lack of obvious obstruction of vessels on surgery, histopathology results indicated that *C. butyricum* isolation from the patient's blood samples resulted from mucosal breakdown and transmigration of these bacteria into the bloodstream. These findings indicated that pathogenic *C. butyricum* infection in the gut resulted in septic shock, leading to ischemia of the small intestine. With regard to the route of infection, in this case, the initial exploratory laparotomy for the stab wound did not involve any bowel injuries; therefore it was not related to the development of NEC. We hypothesized that the patient was a *C. butyricum* carrier, and treatment with several antibiotics for methicillin‐resistant *Staphylococcus aureus* pneumonia might have resulted in the microbial substitution. Nevertheless, the invasion route is still unclear. In addition, a previous report suggested that lactose fermentation is involved in the pathogenesis of NEC.^11^ However, the enteral nutrient administered to this patient did not include lactose. Moreover, the patient had been admitted for 30 days before the development of NEC. During that period, no outbreak of this kind of bacterial infection was observed in our hospital, thus, excluding the possibility of nosocomial infection.^7^ Therefore, the course of *C. butyricum* infection remains unclear.

Regarding the differential diagnosis, we discussed the possibility of the following three diseases. Neutropenic enterocolitis, also known as typhlitis, is confused with NEC; however, our patient's background was completely different to reported cases.^12^ Although its cause is still unclear, it basically occurs in patients with neutropenia, such as leukemic patients receiving chemotherapy. The patient did not have any medical history suggesting neutropenia. In addition, a test for antibodies against HIV yielded negative results. The possibility of non‐occlusive mesenteric ischemia (NOMI) was definitely difficult to rule out, because of similar traits between both diseases: ischemia and infection.^13^ Moreover, segmental and discontinuous necrosis in a region perfused by the superior mesenteric artery can be typically observed in NOMI, although it was difficult to confirm that necrotic and ischemic regions were completely continuous in our case. Nonetheless, if our case was NOMI, the innumerable bacteria propagated in such a short period would have been incongruous, as the patient had no risk factors at the onset, such as cardiac failure, low flow states, multi‐organ dysfunction, or vasopressors.^14^ The probability of ischemic enteritis was low because there was no arteriosclerosis, and it typically occurs in a region perfused by the inferior mesenteric artery. Thus, based on the above reasons, we diagnosed NEC.

Regarding the prevention of adult NEC, a clonal strain was found to be circulating in neonatal intensive care unit, during an outbreak of NEC in preterm neonates.^5^^,^ 7 ^,^ 15 Therefore, it is necessary for medical and nursing staff to control infection effectively, even in adult care units. In addition, although prediction is difficult, a risk for developing NEC, owing to microbial substitution, could be considered during treatment with antibiotics in the same manner as consideration for *C. difficile* colitis.

Limitations of this study include the inability to assess the neurogenic symptoms (vision impairment and dysphagia) of the patient, because of his continuous disturbed mental state. Furthermore, gene sequencing to confirm the type of strain isolated could not be carried out.

## Conclusion

We present a rare case of successful management of a hospitalized elderly patient with NEC associated with *C. butyricum*. The present case suggests that hospitalized adults who receive antibiotic therapy carry a risk of critical illness associated with pathogenic *C. butyricum*.

## Disclosure

Approval of the research protocol: N/A.

Informed consent: Informed consent was obtained from the subject.

Registry and the registration no. of the study/trial: N/A.

Animal studies: N/A.

Conflict of interest: T.Y. was supported by grants from Kissei Pharmaceutical Co., Ltd. and Nichirei Biosciences Inc. The other authors have no conflict of interest.
